# Shared viral burdens: Molecular evidence of Usutu virus infection and multi-arbovirus exposure in migrant and resident birds at wintering locations in Nigeria

**DOI:** 10.1371/journal.pntd.0013538

**Published:** 2026-08-19

**Authors:** Nnomzie C. Atama, Joy A. Ishong, Cora M. Holicki, Yahkat Barshep, Felicity D. Chandler, Anne van der Linden, Marion P. G. Koopmans, Reina S. Sikkema

**Affiliations:** 1 Viroscience Department, Erasmus Medical Center, Rotterdam, the Netherlands; 2 A.P. Leventis Ornithological Research Institute, Center of Excellence, Plateau State, Nigeria; 3 Department of Zoology, University of Jos, Jos, Nigeria; University of Reunion Island, RÉUNION

## Abstract

**Background:**

West Nile (WNV), Usutu (USUV), and Sindbis (SINV) virus were initially detected in the African region, and subsequently across temperate regions where they were previously absent. Wild birds are primary reservoirs for these arboviruses and are considered major contributors to their global spread through seasonal migration. To understand the transmission dynamics of arboviruses in African wild birds and the potential of migratory birds to spread the viruses at an intercontinental scale, we investigated arboviral infections and exposures in resident and Palearctic migratory birds at wintering locations in Nigeria.

**Methodology/Principal findings:**

Oropharyngeal- and cloacal swabs, feathers and blood were collected from resident and migratory birds at two wintering locations (Amurum and Ngel-Nyaki Forest Reserves). Swabs and feathers were tested using RT-PCR for WNV, USUV and SINV, and blood with ELISA and FRNT_90_ or PRNT_80_ for antibodies. 573 birds were sampled between 2021–2024 across months coinciding with arrival and departure of migratory birds. USUV RNA was detected in 2.6% of feathers including a positive Icterine warbler and a garden warbler sampled prior to spring migration. None of the swabs were positive for viral RNA but neutralizing antibodies to WNV and USUV were detected in 4.5% of birds. SINV antibodies were also found in 34.1% of birds sampled across the wintering locations.

**Conclusions/Significance:**

Our findings provide molecular evidence of USUV RNA detection and serological evidence to USUV, WNV and SINV in migratory and resident birds sampled at overwintering locations in Nigeria. These findings are consistent with the possibility that migratory birds may acquire arboviral infections during their overwintering periods in Africa. In addition, detections of viral RNA through RT-PCR in feathers, but not swabs, suggest feathers may be a suitable alternative sample type for surveillance in the absence of a reliable cold chain. The overall detections in wild birds at these locations highlight the need for further surveillance to define the epidemiology and public health risks of these arboviruses in the region.

## Author summary

Vector-borne viruses like West Nile, Usutu, and Sindbis, were once limited to parts of Africa, but now appear in Europe and other temperate regions. Migratory birds are believed to help spread these viruses across continents. To understand the role of birds in this, we tested resident and migratory birds in Nigeria for evidence of infection. Between 2021 and 2024, we sampled over 570 birds during their winter stay. We found genetic material of Usutu virus in feathers of six birds including two migratory birds just before their return to Europe. While swab tests did not detect active infections, antibodies to these viruses, which indicate past exposure, were found in several birds, especially for Sindbis virus. Our findings show that migratory birds may pick up and possibly carry these viruses across continents. We also showed that feathers may be an alternative practical tool for virus detection in areas without reliable refrigeration, supporting easier and broader surveillance of these viruses in Africa and possibly beyond.

## Introduction

Several mosquito-borne arboviruses, including viruses in the *Flaviviridae* and *Togaviridae* families, are primarily maintained in enzootic transmission cycles between wild birds and mosquitoes [1]; however, others predominantly circulate among humans or mammals [2]. Wild birds have long been identified as reservoir and amplifying hosts for several of these arboviruses, including flaviviruses like West Nile virus (WNV) and Usutu virus (USUV), and alphaviruses like Sindbis virus (SINV) and Western Equine Encephalitis virus (WEEV). Several of these viruses are of veterinary and/or public health significance. For example, WNV has caused many outbreaks in humans and animals [3]. Also, USUV can cause morbidity and mortality in animals, but is considered less pathogenic in humans [4].

Following initial isolation of USUV from field caught *Culex neavei* mosquito in Southern Africa in 1959 [5], the virus was subsequently detected in mosquitoes and vertebrate hosts, mainly birds, across several African countries including Senegal, Central African Republic, Nigeria, Uganda, Burkina Faso, Cote d’Ivoire, and Morocco [6–8]. In Europe, USUV was first detected in 2001 in Vienna, Austria, where it was identified as the cause of mortality in wild birds [9]. Phylogenetic analysis of USUV genomic sequences across Europe revealed similarity and transmission links between USUV strains in Africa and Europe indicating multiple independent introductions through migratory avian hosts [10].

WNV was first isolated in 1939 from the blood of a febrile patient at the West Nile district of Uganda [11]. Since then, the virus has caused sporadic outbreaks in many parts of the world. Genomic evidence shows that some outbreaks in Europe were seeded through migratory birds coming from Africa [12]. WNV has caused high mortality in birds in America, however its impact in birds across Europe has been minimal [13].

SINV is an alphavirus that is transmitted mainly by *Culex* mosquitoes such as *Culex neavei*, *Culex pipiens sensu stricto (s.s.),* and *Culex torrentium* [14,15]. It was first detected in mosquitoes in Egypt in 1952 [16], and has since been found in birds and humans in South Africa [17] and across Northern Europe, and recently in other European countries including Spain, Germany, Romania, and the Netherlands [18–21]. SINV has birds as primary amplifying hosts, and infections in humans often result in mild disease characterized by fever and rash, and sometimes arthralgia, with symptoms persisting for months in a considerable percentage of patients [17].

Within Africa, arboviruses like USUV, WNV, and SINV are maintained in circulation between birds and *Culex* mosquitoes, and infections in local host species seem to cause only minimal fatality. So far, there is scanty evidence for USUV and WNV circulation in birds and humans in Nigeria [6,22–24]. In 1972, in Nigeria, USUV was isolated from passerine bird species including Little greenbul (*Eurillas virens*), a Piping hornbill (*Bycanistes fistulator*), and a Kurrichane thrush (*Turdus libonyana*) [6]. Thereafter, serological evidence for WNV was documented in domestic birds and humans across Nigeria [24–26]. SINV, on the other hand, was only recently detected for the first time in Nigeria from African thrushes (*Turdus pelios*) that were sampled between 2022 and 2023 in a protected area [27].

As a tropical location with diverse habitats and known Important Bird Areas (IBAs), Nigeria hosts a great diversity of birds including endemic species. It serves as an overwintering location for about 253 migratory birds [28]. Several Palearctic migratory bird species that are known reservoirs for arboviruses undertake seasonal migration to overwinter in Nigeria and other parts of Africa [29,30], and their role in the intercontinental spread and emergence of arboviruses is yet to be fully resolved. Therefore, understanding the infection and exposure status of these groups of birds at their overwintering and stopover locations will shed more light on their role in transmission across bird flyways and on the evolution of the virus. Here, we aimed to identify and characterize the circulation and transmission of arboviruses including WNV, USUV, and SINV in African resident and Palearctic migratory birds at two overwintering IBAs in Nigeria – Amurum Forest Reserve (Amurum) and Ngel-Nyaki Forest Reserve (Ngel-Nyaki).

## Methods

### Ethics statement

The handling and sampling of birds was carried out under protocols and permits approved by the Scientific Committee of the A P Leventis Ornithological Research Institute (APLORI), University of Jos, Nigeria. A material transfer agreement (MTA) approved by the Nigerian Federal Ministry of Environment with the FMENV MTA Reference: FOR/ABS PERMIT/12/1 permitted transfer of samples between APLORI and Erasmus Medical Center.

### Study sites

**Fig 1:**
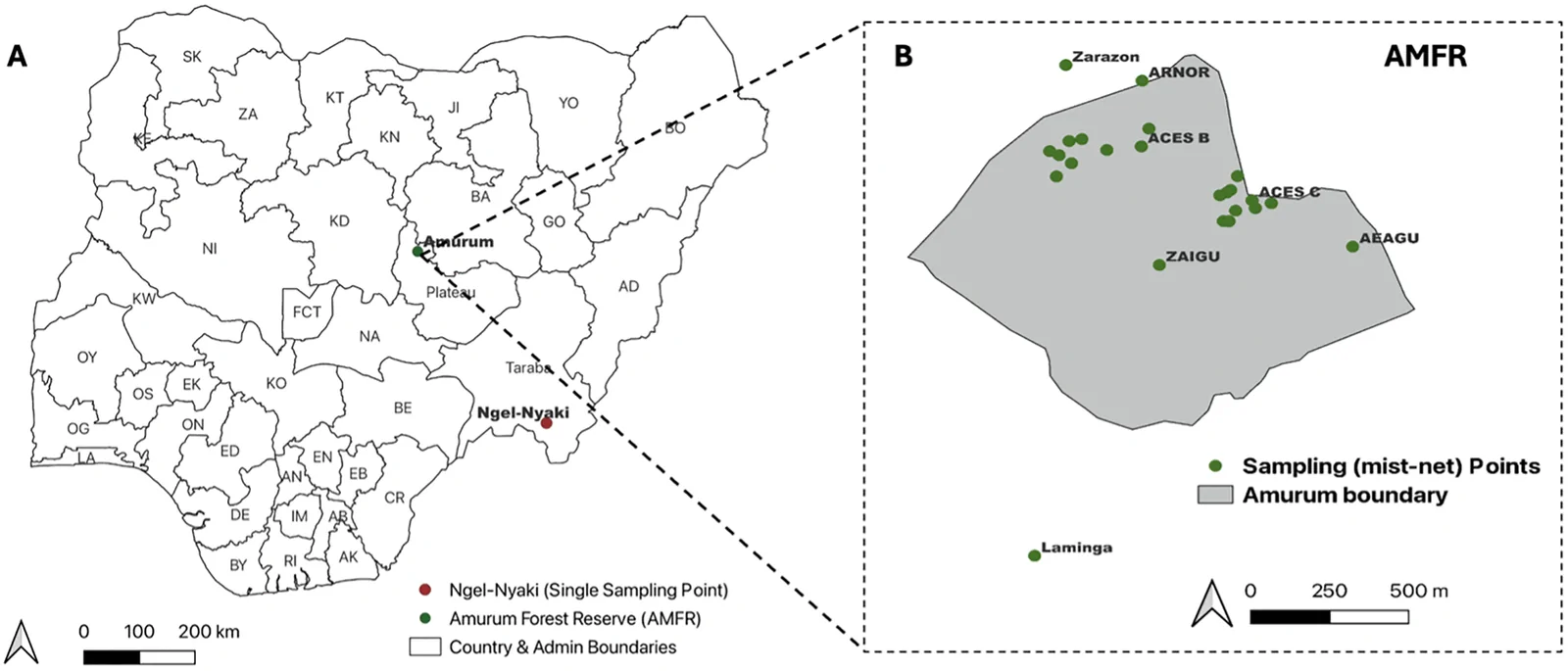
Sampling sites across two overwintering locations in Nigeria. **1A**. Map of Nigeria with geographic placements of Amurum Forest Reserve (AMFR) within Plateau State (green point) and Ngel-Nyaki Forst Reserve (NNFR) in Taraba State (Red). *Base map source* [35]. 1**B**. Expanded boundary map of Amurum Forest Reserve (AMFR) showing the different mist-netting locations (green points) across the Reserve and its environs. NB: AMFR shapefiles and boundary maps were provided by A. P. Leventis Ornithological Research Institute (APLORI) via QGIS Project Case Studies; produced with institutional permission. Sampling was conducted at constant effort sampling (CES) sites within the reserve including CESB, CESC, Zainab’s Gully (ZAIGU), Eastern Gully (AEAGU), North of the Reserve (ARNOR), and at outside locations bordering the reserve - Laminga village (Laminga) and Zarazon. NB. Sampling at Ngel-Nyaki (NNFR), was conducted at one location (with the exact geolocation shown on **1A**).

The study was conducted in Nigeria at two protected areas – within and around the vicinity of Amurum Forest Reserve (Amurum) and at Ngel-Nyaki Forest Reserve (Ngel-Nyaki). Amurum is in the North-Eastern part of Plateau State in North-Central Nigeria (9°53′N, 8°59′E) covering an area of 300 hectares and composed of granitic outcrops and patchy grasslands with interspersed gallery forests [31]. Ngel-Nyaki, however, is a montane forest area in the Mambila plateau located 60 km west of Gashaka-Gumti National Park (7  .08° N, 11.13 °E). It extends to about 45 km^2^ with 7 – 7.2 km^2^ of dense montane forest and an 85% shrubland cover. Its wet season lasts from March to November with an average annual rainfall of about 2,000 mm [32] (Fig 1A and 1B). Both locations are protected areas rich in biodiversity and avifauna and are recognized as IBAs according to Birdlife International [28]. They both also serve as breeding locations for resident and intra-African migratory birds, and as overwintering locations for Palearctic migratory birds [28] that mostly arrive the wintering locations around September/October and depart in spring (March/April) [33,34].

### Bird sampling and sample collection

As part of APLORI’s Constant Effort ringing scheme [36] conducted across Constant Effort Sites (CES) in and around Amurum and at Ngel-Nyaki, wild birds including African resident and Palearctic migratory birds, were mist-netted, ringed, and sampled (Fig 1A and 1B). In Amurum, sampling was conducted across dry and wet seasons, between March - December 2021, February - April 2022, and April - December 2024. At Ngel-Nyaki, however, samples were only collected once, in March of 2022. Birds were sampled across seven sites within and around Amurum, and at one location in Ngel-Nyaki (Fig 1B). Sampling was conducted to tally with periods spent by migratory birds at the wintering locations, based on over 20 years of observational and ringing data on migratory birds through APLORI Bird Ringing (APBRING) Scheme [36]. Birds caught were identified to species, aged (from plumage as years relative to hatch year), and sexed when possible. Ringed birds were sampled by collecting swabs (oropharyngeal and/or cloaca) in 1.5 mL RNA Later (ThermoFisher Scientific, Vilnius, Lithuania). For smaller birds, only oropharyngeal swabs were collected, whereas in the larger birds, cloacal swabs were also taken and pooled with throat swabs. Wing or breast feathers were collected, and blood collected by brachial venipuncture when possible. Biometric measures including wing length, body mass, fat score, and moult score were taken. In 2024, only feather samples were collected from sampled birds at Amurum. All samples were stored at +4^o^C for approximately two weeks before being transferred to -80^o^C and later shipped on dry ice to the WHO arbovirus reference laboratory at Viroscience Department of Erasmus Medical Center in Rotterdam, The Netherlands for molecular and serological analyses.

### Molecular analysis

Swab samples were prepared by first transferring swabs from RNA later into 2 mL sample tubes containing virus transport medium, vortexed, and centrifuged at 10,000 xg for 1 minute. Total nucleic acid (tNA) was extracted by eluting 400 µL of swab material in 600 µL of Roche external lysis buffer and processed using an MP96 MagNA pure LC instrument (Roche Diagnostics GmbH, Mannheim, Germany). The larger wing feather samples were processed by splitting 1 cm of the lower half of the feather calamus and homogenizing in 350 µL Roche tissue lysis buffer plus ¼” ceramic beads using a FastPrep-24 5G tissue Magnilizer (M.P. Biomedicals, LLC, California, USA). The smaller breast feathers were, however, homogenized whole at 60 sec; 6.5 m/s as previously described [37].

Extracted RNA (tNA) was tested on screening and confirmatory RT-PCRs for WNV, USUV, tick-borne encephalitis virus (TBEV), Japanese encephalitis virus (JEV), and SINV targeting different genomic regions of each of the viruses with primers and probes as described previously [38]. RNA from swabs were tested in pools of five, while feather samples were tested individually. Samples were considered positive only if they had cycle threshold (C_T_) values < 40 in both the screening and confirmatory PCR assays (s2). When C_T_-values of positive samples were too high to sequence (> 32), blood clots of positive birds, which were obtained after separation of serum, were also tested. Adapting from a previously described protocol [39], a pinhead-size of blood clot was added to 600 µL tissue lysis buffer and 6 µL β-mercaptoethanol with 5 mm ceramic beads and homogenized at 4 m/s for 20 sec using the FastPrep-24 tissue Magnalizer. Then 50 µL of the supernatant was eluted in 150 µL binding/lysis buffer and 400 µL PBS to extract RNA based on a manual column extraction protocol using High Pure RNA Isolation kit (Roche Diagnostic GmbH, Mannheim, Germany) according to manufacturer’s instruction. In general, samples that were positive on both screening and confirmatory PCRs were also tested on hemi-nested and nested pan-PCRs designed for flaviviruses and alphaviruses targeting conserved regions of NS5 gene and NSP4 gene, respectively, as previously described [40,41]. The products of the pan-PCRs were then submitted for sequencing using a Sanger sequencing approach.

### Serological analysis

Blood samples were centrifuged at 10,000 xg for 5 minutes to collect serum. Most of the blood samples were stored longer before centrifugation which resulted in impure sera with red blood cell content. Collected supernatant were stored under -80^o^C until they were analyzed.

#### ELISA.

All serum samples were screened for binding antibodies to the domain III of the E protein of WNV using a commercial blocking ELISA (*INGEZIM West Nile COMPAC – Eurofins Technologies, Madrid, Spain*) according to the manufacturer’s instructions, where an inhibition percentage (IP) ≥ 40% was termed positive, IP > 30% or < 40% as doubtful and ≤ 30% as negative. The ELISA kit has the potential to detect cross-reactivity with USUV and was thus used to also capture antibodies to USUV if present [42,43]. Samples reactive on ELISA (positive or doubtful) were further tested for neutralizing antibodies using a focus reduction neutralization test (FRNT) to differentiate positive from doubtful ELISA results and classify reactivity as either to WNV or USUV.

#### WNV-USUV FRNT.

The ELISA positive samples were tested for WNV and USUV neutralizing antibodies using in-house validated assays (WNV- and USUV-FRNT) that assess presence of neutralizing antibodies based on a 90% reduction in foci (cluster of infected cells) on a confluent monolayer of Vero cells for WNV and USUV, respectively [44]. Initial testing demonstrated that the FRNT read-out was hampered by cell debris from the poor-quality sera. Therefore, an additional washing step with subsequent addition of infection medium (DMEM supplemented with FBS) was added to the protocol after a 2-hour incubation step of the serum-virus mix on the cell monolayer. The plates were then incubated for 24 hours at 37^o^C following standard protocol. A sample was defined as seropositive for WNV or USUV if it showed neutralization at titer ≥ 80 (for WNV) or ≥ 160 (for USUV) and a 4-fold difference in titer between WNV and USUV or vice-versa.

#### SINV PRNT.

Due to limited serum volumes, only samples negative in the WNV ELISA, which still had serum left, were tested for neutralizing antibodies to SINV (strain 09-M-991-1 from Sweden, Accession number: KF297653) using a plaque reduction neutralization test (PRNT) on a Vero ATCC CCL-81 cell line [18,45]. Samples that were positive in the pre-screening (1:20 dilution) were serially diluted from 1:20–1:640. Samples were dimmed positive if they showed 80% reduction of plaque counts compared to a virus control in the pre-screening and end-titration. Due in part to the poor quality of the sera, individual samples were pre-screened or end-titrated a second time, and the average titer was calculated. Samples that were too haemolytic for the read-out were excluded.

### Data analysis

Data management, analysis, and visualization of graphs were done using Microsoft Excel v16.95.4 and RStudio v2024.12.1.563 [46]. SINV seroprevalence was estimated as proportion of positive individuals among the total tested, with exact binomial 95% confidence intervals (CIs) calculated using the Wilson score method [47]. We fitted a mixed-effects logistic regression model using the lme4 package in R, with SINV seropositive status (positive/negative) as dependent variable. Migratory status and season were included as fixed effects, while species and sampling location were treated as random intercepts to account for clustering and reduce collinearity. Performance package in R was used to assess model performance [48].

## Results

573 birds of 59 species were sampled in 2021, 2022, and 2024. In 2021 and 2022, wild birds were sampled for swabs (oropharyngeal and/or cloaca), feather, and blood, whereas in 2024 only feather samples were collected. Most samples were collected from birds at Amurum and only 12 of the 573 birds were sampled at Ngel-Nyaki (Fig 1A). 51.1% (293) of the sampled birds were migratory birds comprising 17 species, while the others were African resident birds. 52.5% (301) of the samples were collected during the wet seasons of the sampling years (between late April to early October) when mosquito abundance is highest [49]. Swabs were collected from 464 birds, feathers from 229, and blood from 264 birds.

### Molecular detections

All swabs were negative for arboviruses in RT-PCR. However, USUV RNA was detected by RT-PCR in feathers of six birds at a prevalence of 2.6% (6/229; 95% CI: 1.2 - 5.6). None of the feathers were positive for WNV, TBEV, or JEV. A feather sample from a Spotted flycatcher (*Muscicapa striata*) tested positive for SINV on a first PCR (C_T_ 35) but could not be confirmed using a second PCR that targeted a different region of the SINV genome. None of the blood clots from the USUV positive feathers tested positive for USUV RNA. The six USUV positive birds included three African resident birds (two African thrushes; *Turdus pelios* and a Black-necked weaver; *Ploceus nigricollis*), and three Palearctic migrant birds (Tree pipit; *Anthus trivialis*, Icterine warbler; *Hippolais icterina*, and Garden warbler; *Sylvia borin*). The USUV positive African thrushes, Black-necked weaver, and Tree pipit were sampled during the dry season (between December 2021 and February 2022). The positive Icterine warbler and Garden warbler were however sampled during the wet season; in April 2022 and October 2024, respectively. The C_T_-values of the positive birds ranged between 34.9 – 37.1 (median C_T_ 35.6) (Supplemental S1 Table). Except the positive garden warbler, which was sampled at Laminga, a village bordering Amurum, all other RNA positive birds were trapped and sampled within the reserve, at the Constant Effort Site B (ACESB) – a location where 57.9% (332) of the total birds were sampled (Fig 1B).

All sequencing attempts failed due to low viral loads in positive samples and no genomic sequences were generated from any of the USUV positives.

### Serologic detections

**Fig 2:**
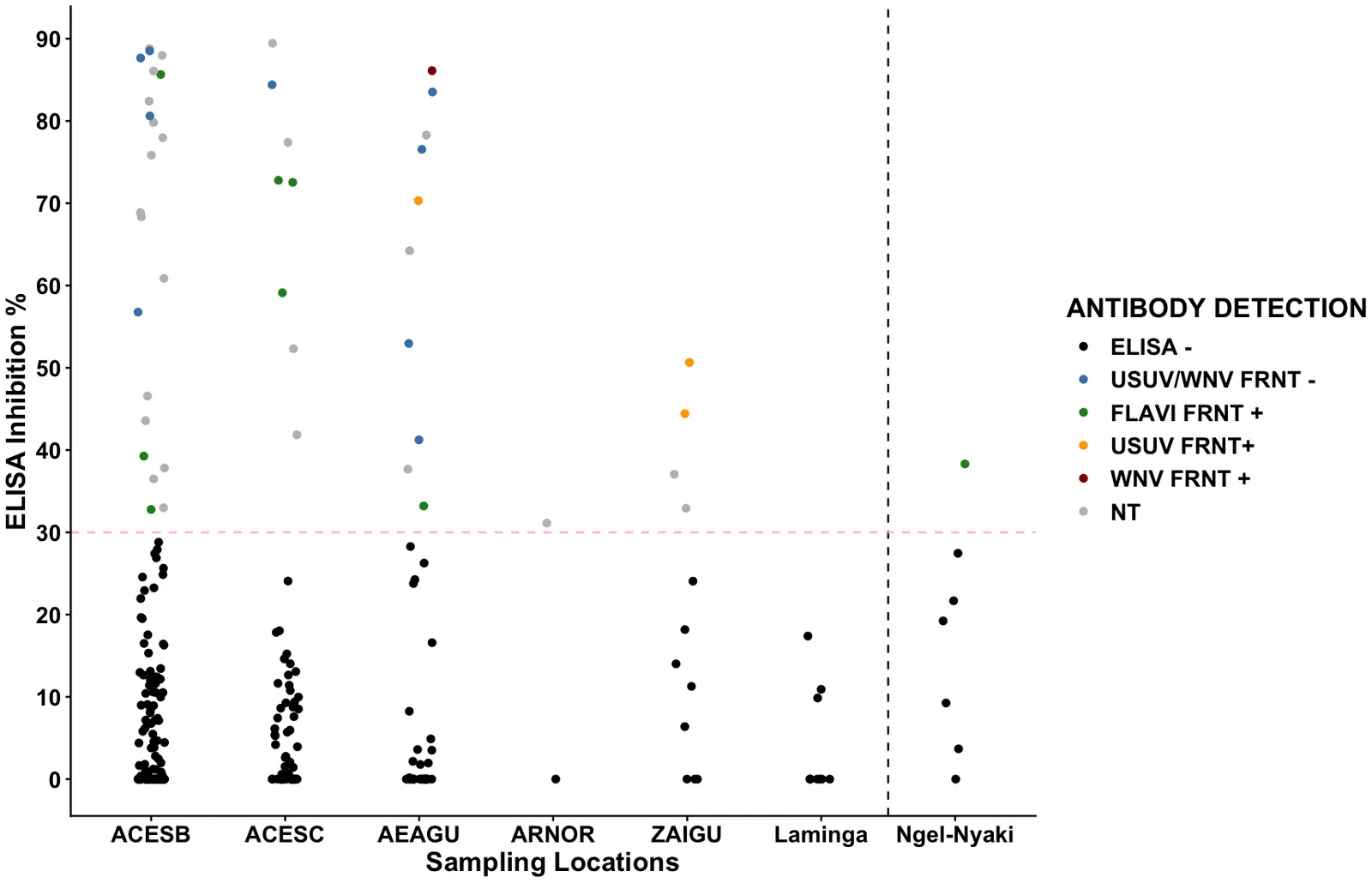
Detections and locations of WNV, USUV, and Orthoflavivirus (FLAVI) seropositive birds. *Virus-specific antibody responses were confirmed via FRNT*_*90*_ *for WNV (red), USUV (orange), and FLAVI (green) from ELISA reactive samples (ELISA Inhibition % ≥ 30). ELISA reactive samples with insufficient volume could not be further tested on FRNT*_*90*_ *(NT)*.

46 out of 264 (17.4%) serum samples were reactive on the ELISA, with 35 positives (IP ≥40%) and 11 doubtful (IP > 30% and <40%). 25 of the 46 reactive samples (18 positives and 7 doubtful) had insufficient sera volume remaining and were not tested for neutralizing antibodies on FRNT. An overall 4.5% (12/264; 95% CI: 2.6 - 7.8) of the birds had neutralizing antibodies to flaviviruses. Four out of 21 ELISA positive/doubtful samples were confirmed to have neutralizing antibodies to either WNV (n = 1) or USUV (n = 3). Eight other samples (8/21) which could not be differentiated based on 4-fold titer difference (n = 3) or that were neutralizing on either the WNV or USUV FRNT but had insufficient serum volume to test on both assays (n = 5) were classified as Orthoflavivirus positive (FLAVI) (Table 1; Fig 2). The WNV- and USUV-antibody positive birds were all African thrushes (resident bird species) sampled in the wet season of 2021. The FLAVI positives, however, included resident birds (three African thrushes, a blackcap babbler; *Turdoides reinwardtii*, a yellow-crowned gonolek; *Laniarius barbarus*, and a laughing dove; *Spilopelia senegalensis*) and migratory birds (two garden warblers). All the positive birds were sampled across four CES (ZAIGU, AEAGU, ACESB and ACESC) within Amurum, except one FLAVI-positive garden warbler that was sampled at Ngel-Nyaki (Table 1 and Fig 2).

**Table 1:** ELISA positive samples across Afro-Palearctic migrant and African resident birds confirmed on FRNT for neutralizing antibodies to WNV, USUV, or Orthoflavivirus (FLAVI). NB. ^☨^ are samples that were seropositive on the USUV FRNT but could not be retested with washing due to insufficient volumes and were thus marked as Orthoflavivirus-positive (FLAVI).

Bird ID	Species	Ring No.	Migratory status	Sampling site	Sampling year	Blocking ELISA		FRNT_90_		
						Inhibition Percentage (IP%)	Results	WNV Antibody Titers	USUV Antibody Titers	Confirmed Antibody Status
22NG0286	African Thrush	4A61014	Resident	ZAIGU	2021	50.6	POS	160	640	USUV
22NG0287	African Thrush	4A61013	Resident	ZAIGU	2021	44.4	POS	10	640	USUV
22NG0260	African Thrush	4A61020	Resident	AEAGU	2021	33.2	DOUBTFUL	160	NT	FLAVI
22NG0268	African Thrush	4A62984	Resident	AEAGU	2021	86.1	POS	320	20	WNV
22NG0297	African Thrush	4A61027	Resident	AEAGU	2021	70.3	POS	10	320	USUV
22NG0338	African Thrush	4A62267	Resident	ACESC	2021	59.1	POS	10	320 ^☨^	FLAVI
22NG0370	African Thrush	4A61831	Resident	ACESC	2021	72.8	POS	10	320 ^☨^	FLAVI
21NG014	Blackcap Babbler	DH05223	Resident	ACESB	2021	85.6	POS	160	NT	FLAVI
21NG0076	Garden Warbler	Y24390	Migrant	Ngel-Nyaki	2022	38.3	DOUBTFUL	NT	160	FLAVI
21NG0064	Yellow-crowned Gonolek	4A62656	Resident	ACESC	2022	72.5	POS	80	NT	FLAVI
21NG0015B	Laughing Dove	D96119	Resident	ACESB	2022	32.8	DOUBTFUL	20	320 ^☨^	FLAVI
21NG0165	Garden Warbler	AR8057	Migrant	ACESB	2022	39.3	DOUBTFUL	160	NT	FLAVI

Functional (neutralizing) antibodies were also detected in 45 of 132 (34.1%, 95% CI: 26.6 - 42.5) birds tested via SINV PRNT_80_. Antibodies to SINV were found in 23 bird species, representing eight migratory species groups and 15 resident species (Table 2). Besides two speckled mousebirds; *Colius striatus* (resident birds) and a blackcap; *Sylvia atricapilla* (a migratory bird) which were sampled at Ngel-Nyaki, all other SINV antibody-positive birds were sampled across different sites in Amurum. SINV antibody titers in both migratory and resident birds ranged between 20–320 (median titer 40). An estimated seroprevalence of 46.9% (95% CI: 30.6 - 63.9) was recorded in migratory birds, and 30.0% (95% CI: 21.8 - 39.7) in resident birds. In the mixed-effects logistic regression model, neither migratory status of the birds nor season showed statistically significant associations with SINV seropositivity. Resident birds had lower odds of SINV antibody detection compared to migratory birds (OR = 0.52, 95% CI: 0.19 – 1.47, p = 0.22), although this effect did not reach significance. Similarly, individuals sampled during the wet season, exhibited higher odds of seropositivity compared to those sampled during the dry season (OR = 1.61, 95% CI: 0.63 – 4.09, p = 0.32), but again, this difference was not significant. The wide confidence intervals indicate considerable uncertainty, likely due to limited sample sizes and variability among species and locations.

**Table 2:** Overview of SINV antibody-positive Afro-palearctic migrant and African resident bird species.

Species					
Palearctic migrant	Sampling sites	Seasons	Months	Years	SINV PRNT_80_ Pos/Tested (~ %)
Blackcap	Ngel-Nyaki	dry	Mar	2022	1/2 (50)
Common Whitethroat	AEAGU	dry	Mar	2022	1/2 (50)
European Nightjar	ACESB	wet	Apr	2022	1/1 (100)
Garden Warbler	ACESB, ACESC	wet	Apr, Mar	2021- 2022	4/6 (67)
Icterine Warbler	ACESB, ACESC	wet	Apr	2021	2/2 (100)
Pied Flycatcher	ACESC	wet	Oct	2021	2/5 (40)
Spotted Flycatcher	ACESB, ACESC	wet	Apr	2021	3/8 (38)
**African Resident**					
Adamawa turtle dove	ACESB	dry	Feb	2022	1/12 (8)
African Thrush	ACESB, ACESC, AEAGU	dry, wet	Feb, Mar, Apr, Dec	2021-2022	9/18 (50)
Common Bulbul	ACESC, AEAGU, ZAIGU	dry, wet	Apr, Mar, Dec	2021, 2022	3/6 (50)
Mocking Cliff Chat	ACESC	wet	Oct	2021	1/2 (50)
Moustached Grass Warbler	ACESC	wet	Apr	2022	1/1 (100)
Northern Red Bishop	ACESB	wet	Apr	2022	1/1 (100)
Orange-breasted Bushshrike	ACESC	dry	Feb	2022	1/1 (100)
Senegal Coucal	ACESB	dry	Feb	2022	1/1 (100)
Snowy-crowned Robin Chat	ACESB, ACESC	wet	Apr	2022	2/4(50)
Speckled Mousebird	AEAGU, Ngel-Nyaki	dry, wet	Apr, Mar	2021- 2022	3/8 (38)
Village Weaver	ACESB, ACESC, AEAGU	dry, wet	Feb, Apr	2021- 2022	4/21 (19)
Violet-backed Starling	Laminga	wet	May	2021	1/1 (100)
Yellow-billed Shrike	ACESB	dry	Dec	2021	1/1 (100)
Yellow-mantled Widowbird	ACESB	dry	Feb	2022	1/1 (100)
Yellow-throated Greenbul	Laminga	wet	May	2021	1/3 (33)

## Discussion

Here, we set out to study the circulation and transmission of WNV, USUV, and SINV in African resident and Palearctic migratory birds at two overwintering locations in Nigeria. Our study provides molecular evidence of USUV RNA detection in resident and migratory birds and serological evidence of exposure to multiple arboviruses in the study areas in Nigeria. USUV RNA was identified in feather samples of both resident and migratory bird species. This likely reflects overlapping ecological niches and exposure to common vector populations at wintering sites in the same region [50]. Importantly, one of the USUV-positive birds in our study was an Icterine warbler (a Palearctic migrant) which was sampled in April, prior to spring migration (late February to late June as described by Jonzén *et al.* (2007) [51]. The detection of USUV RNA in migratory species in our study suggests that these birds may contribute to long distance dispersal of the virus, although the present study was not designed to directly assess transmission across flyways.

All positive samples were confirmed by two independent RT-PCR assays targeting different genomic regions, we were unable to recover sequence data or confirm the presence of infectious virus through culture because of the low viral loads. Therefore, the findings should be interpreted as evidence of USUV RNA detection consistent with recent infection rather than definitive proof of active transmission.

An earlier analysis based on species chorotypes that defined the migratory patterns of birds in Africa and Europe already classified the Icterine warbler as a potential vector of WNV across both continents [52]. The detection of viral RNA in the Garden warbler sampled in October of 2024, corresponded to a period upon arrival at wintering location, but whether the individual returned infected or picked up infection upon arrival or along migratory route could not be ascertained. While no sequences were obtained in our study to confirm this for USUV or WNV, our findings align with previous suggestions of flyway-mediated transmission based on phylogenetic and quantitative models [12,52–54].

USUV RNA could not be detected in the swabs, whereas the feathers of some swab-negative birds were positive. This may have resulted from degradation of viral RNA during storage and transport of the swabs. Prior to shipping, the swabs in VTM were kept for approximately 2 weeks under +4^o^C and subsequently in -80^o^C before been shipped on dry-ice. The temperature fluctuations may have degraded the quality of viral RNA if present in the samples. Long persistence of viral RNA in feathers of infected birds has previously been documented for USUV and Influenza viruses [37,55]. Alternatively, differences in tissue-specific persistence of viral RNA, or the stage of infection at sampling may have contributed to the difference in detection sensitivity. Our detection of USUV RNA in feathers from birds that tested negative on swabs suggest that feathers may represent a useful alternative sample type for arbovirus surveillance, especially under remote field conditions where sustained cold storage cannot be guaranteed. Other studies have demonstrated the sensitivity of feathers relative to swabs for USUV RNA detection [37], however, in our current study we did not assess storage conditions and sample degradation.

Antibodies to both WNV and USUV were also detected in resident and migratory birds, with no significant difference in prevalence or antibody titers, suggesting comparable exposure risk across both groups. The concurrent detection of viral RNA and neutralizing antibodies in African thrushes indicates their potential role as reservoirs for USUV, and possibly WNV, in the region. USUV was previously identified in a South African thrush species - a Kurrichane thrush (*Turdus libonyana*) - which was reported to have been sampled in Nigeria [56]. Our findings in African thrushes (*Turdus pelios*) further supports the susceptibility of thrushes to infection in the region. Susceptible host species often share ecological niches [57]. The Song thrush (*Turdus philomelos*), a closely related species in Europe, occupies a similar ecological niche and has repeatedly been reported as a susceptible host of USUV [58,59]. This parallel suggests that members of the genus *Turdus* may share ecological and biological traits that are relevant for USUV maintenance, although the specific reservoir potential of African thrushes requires further investigation.

Evidence of SINV infection was detected through a screening PCR in a spotted flycatcher, a migratory bird; however, this could not be confirmed by a subsequent confirmatory PCR. Nevertheless, a very recent study at the same sampling location - Amurum, detected SINV in African thrushes [27], confirming enzootic circulation of the virus and the risk of infection to migratory species overwintering at the location. In addition, we found that antibodies to SINV were widely detected across several African resident and European migratory birds in the region, including three out of eight spotted flycatchers. Similarly, SINV antibodies were found in other migratory birds sampled at Amurum. Moreso, antibodies to SINV were detected in migratory and resident birds sampled at Ngel-Nyaki, a location about 300 kilometres away from Amurum, suggesting a broader geographic risk for SINV in Nigeria. Since we neither tested nor compared antibody cross-reactivity to other alphaviruses within the Sindbis virus antigenic group, such as Babanki virus, and to a lesser extent, Semliki Forest virus [60,61], the possibility of mixed infections or misdiagnosis due to cross-reactivity cannot be entirely ruled out. In the SINV antibody positive migratory birds, the presence of viral RNA could not be confirmed, thus limiting our ability to infer the true source of infections or determine if these individuals were infected at their wintering locations or elsewhere. In addition, future studies would benefit from integrating bird, human, and entomological surveillance. Concurrent testing of mosquitoes and birds could provide stronger evidence of local transmission, identify the vector species involved, and improve understanding of the ecological drivers of arbovirus circulation at these overwintering sites.

## Conclusion

In summary, our findings indicate that migratory birds can become infected with USUV, and potentially with SINV during their overwintering periods in sub-Saharan Africa. Although we did not detect viral RNA in swabs or blood samples, the molecular detection of USUV in feathers suggests possible infections of migratory birds at their wintering locations. Due to poor quality of sera, most of the ELISA positive samples could not be read out in the neutralization assays for functional antibodies, possibly resulting in an underestimation of the number of WNV and USUV infected birds at the study locations. Also, only the ELISA negative samples were tested for SINV, lowering the sample size for our SINV antibody survey. The molecular evidence for USUV, and exposures to WNV and SINV in wild birds in this study, coupled with earlier published molecular evidence for circulation of SINV at one of our sampled locations [27], highlights a potentially wider arbovirus risk in Nigeria that warrants surveillance to understand the epidemiological patterns and public health risks due to possible spillovers. In addition, real-time testing of birds in the region could also maximize the potential of detecting ongoing infections and aid the recovery of sufficient viral genetic material to sequence and characterize viral strains and provide strong evidence for viral transmission across bird flyways.

## Supporting information

S1 Table Overview of Usutu virus positive birds sampled around the vicinity of Amurum Forest Reserve, Laminga, Nigeria between 2021 and 2024. (DOCX)

S2 Table Primers- and probe sequences used for screening and confirmatory testing for Usutu virus. (DOCX)
