# Supplementary material for: Shared viral burdens: Molecular evidence of Usutu virus infection and multi-arbovirus exposure in migrant and resident birds at wintering locations in Nigeria
Source: PLoS Negl Trop Dis. 2026 Aug 19;20(8):e0013538. doi: 10.1371/journal.pntd.0013538 (PMC13502912; doi:10.1371/journal.pntd.0013538)
Supplement: Supplementary file 1 [file pntd.0013538.s001.docx]

**Shared viral burdens: Molecular evidence of Usutu virus infection and multi-arbovirus exposure in migrant and resident birds at wintering locations in Nigeria**

Nnomzie C Atama ^1^, Joy A Ishong ^2^, Cora M Holicki ^1^, Yahkat Barshep ^2,3^, Felicity D Chandler ^1^, Anne van der Linden^1^, Marion P G Koopmans ^1¶^, Reina S Sikkema^1¶^

**Supplemental Table**

**S1. Overview of Usutu virus positive birds sampled around the vicinity of Amurum Forest Reserve, Laminga, Nigeria between 2021 and 2024.**

| **Bird ID** | **Ring Number** | **Species** | **Sampling Date** | **Sampling Location** | **USUV Screening Assay (Ct)** | **USUV Confirmatory Assay (Ct)** |
| --- | --- | --- | --- | --- | --- | --- |
| 21NG007 | 4A81184 | African Thrush | 7-Dec-21 | ACESB | 34.72 | 34.94 |
| 21NG0051 | AR67199 | Tree Pipit | 22-Feb-22 | ACESB | 37.07 | 35.51 |
| 21NG0009 | CA01646 | Black-necked Weaver | 24-Feb-22 | ACESB | 37.85 | 35.29 |
| 21NG0021 | 4A81216 | African Thrush | 24-Feb-22 | ACESB | 37.33 | 35.7 |
| 21NG0026B | AR68033 | Icterine Warbler | 25-Apr-22 | ACESB | 37.15 | 37.14 |
| 21NG008B | AS75070 | Garden warbler | 4-Oct-24 | Laminga | 37.25 | 35.72 |
