# Supplementary material for: Shared viral burdens: Molecular evidence of Usutu virus infection and multi-arbovirus exposure in migrant and resident birds at wintering locations in Nigeria
Source: PLoS Negl Trop Dis. 2026 Aug 19;20(8):e0013538. doi: 10.1371/journal.pntd.0013538 (PMC13502912; doi:10.1371/journal.pntd.0013538)
Supplement: Supplementary file 2 [file pntd.0013538.s002.docx]

**Shared viral burdens: Molecular evidence of Usutu virus infection and multi-arbovirus exposure in migrant and resident birds at wintering locations in Nigeria**

Nnomzie C Atama ^1^, Joy A Ishong ^2^, Cora M Holicki ^1^, Yahkat Barshep ^2,3^, Felicity D Chandler ^1^, Anne van der Linden^1^, Marion P G Koopmans ^1¶^, Reina S Sikkema^1¶^

**S2. Primers- and probe sequences used for screening and confirmatory testing for Usutu virus**

| **Assay** | **Region** | **Forward primer** | **Reverse primer** | **Probe** | **Reference** |
| --- | --- | --- | --- | --- | --- |
| Screening | NS5 | GCCAATGCCCTGCACTTT | TCCCGAGGAGGGTTTCCA | CGATGTCCAAGGTCAGAAAAGACGTGC | Nikolay et al., 2014 |
| Confirmatory | NS5 | CAAAGCTGGACAGACATCCCTTAC | CGTAGATGTTTTCAGCCCACGT | AAGACATATGGTGTGGAAGCCTGATAGGCA | Jöst et al., 2011 |

NB: The PCR primers and probes for the screening and confirmatory PCR were designed to amplify 103 bp and 91 amplicons of the NS5 region of USUV respectively.

**References**

Jöst, H., Bialonski, A., Maus, D., Sambri, V., Eiden, M., Groschup, M.H., Günther, S., Becker, N., Schmidt-Chanasit, J., 2011. Isolation of Usutu virus in Germany. American Journal of Tropical Medicine and Hygiene 85, 551–553. https://doi.org/10.4269/ajtmh.2011.11-0248

Nikolay, B., Weidmann, M., Dupressoir, A., Faye, O., Boye, C.S., Diallo, M., Sall, A.A., 2014. Development of a Usutu virus specific real-time reverse transcription PCR assay based on sequenced strains from Africa and Europe. Journal of Virological Methods 197, 51–54. https://doi.org/10.1016/j.jviromet.2013.08.039
