# Supplementary material for: Shared viral burdens: Molecular evidence of Usutu virus infection and multi-arbovirus exposure in migrant and resident birds at wintering locations in Nigeria
Source: PLoS Negl Trop Dis. 2026 Aug 19;20(8):e0013538. doi: 10.1371/journal.pntd.0013538 (PMC13502912; doi:10.1371/journal.pntd.0013538)
Supplement: Supplementary file 3 [file pntd.0013538.s004.docx]

Viroscience, Erasmus MC

Rotterdam, the Netherlands.

21-06-2026.

The Editor

PLoS Neglected Tropical Diseases

Dear Editor,

**RESPONSE TO REVIEWERS’ COMMENTS**

We appreciate your consideration of our manuscript with Submission (PNTD-D-25-01602) - [EMID:b7d628e2ac398162] and acknowledge your valid suggestions and the important points raised by the independent reviewer aimed at improving the quality of our manuscript.

We have carefully considered the all queries and suggestions and tried to respond to each as best as we could.

Kindly see below the reviewers’ comments (in blue) and our corresponding responses (in black).

Kind regards,

Nnomzie Atama

(On behalf of all authors)

**Reviewers' comments:**

Reviewer #1: Thank you for inviting me to review 'Shared viral burdens: Evidence of active Usutu virus circulation and multi-arbovirus exposure in migrant and resident birds at wintering locations in Nigeria' by Atama et al. The manuscript was very interesting, study well designed and very well written. Overall the manuscript warrants publication and used appropriate statistical analyses throughout.
I really only have a couple of very minor edits to suggest:

**Reviewer’s comment 1**
L41: Should 'was' be 'were'

**Response**

We thank the reviewer for this suggestion. To improve readability and conform to common scientific usage, we have revised the sentence to read as "None of the swabs were positive for viral RNA..."

**Reviewer’s comment**
L105-107: you dont specify that these were sampled in Nigeria, its implied, but might not be obvious

**Response**

We thank the reviewer for making this critical observation. We have revised the sentence to address that ambuguity (L109, 113 - 114).

**Reviewer’s comment**
L108: When was SINV detected, if someone reads this paper in 20 years the detection wouldn't be recent, so best to be specific

**Response**

To address the reviewer’s valid concern on periodicity of SINV detection years from now, we have revised the statement to include the periods when the SINV-positive birds (African thrushes) were sampled (L113 – 114).

**Reviewer’s comment**
L112: 253 migratory bird 'species'. Just to clarify that it isn't just a home for 253 birds.

**Response**

We thank the reviewer for identifying this ambiguity. The text has been revised to indicate that Nigeria hosts approximately 253 migratory bird species (L116).

**Reviewer’s comment**
L175: You say swabs were shortly stored under 4oC. Can you be more specific, what was the window where they were out of cold storage, how many hours?

**Response**

The samples were stored at 4°C for about 2 weeks before long-term storage at −80°C. We have also included this detail in the manuscript to give clarity (L180 - 181).

**Reviewer’s comment**
L193: We typically use ct 35 as a cut off, due to inability to sequence and corroborate the PCR. Can the authors confirm that the 6 positive USUV feathers were positive on both the screening and confirmatory PCR assays, and can they provide cts for both assays in the results section. This will provide important evidence for one of their main findings. Moreover, if the authors could provide the number of bp each assay should recover that would be beneficial.

**Response**

We thank the reviewer for this important suggestion. All six USUV-positive feather samples were positive in both the screening and confirmatory RT-PCR assays. We have now included the Ct values for both assays in a supplementary table (S1) and have provided referenced information on the target regions, primers and propes for each PCR, and the expected amplicon lengths of the screening and confirmatory assays provided in the Suppl;ementary Table S2.

**Reviewer’s comment**
L334: The low number of positives and very late cts dont immediately suggest active circulation. These could have been historic infections picked up from other localities prior to sampling in Nigeria. Moreover, given the broad geographic range of these arboviruses, the serological results could also be due to historic exposure in different areas. I agree transmission is likely to occur in Nigeria, but could the authors contextualise these findings appropriately throughout the ms, with some caveats.

**Response**

We thank the reviewer for this critical observation. We agree that the relatively low number of RT-PCR-positive birds, the high Ct values, and the absence of sequence confirmation or virus isolation limit the strength of inference regarding ongoing local transmission. In addition, we acknowledge that the serological findings may reflect previous exposure acquired at locations other than the sampling sites, particularly in the migratory species. We have therefore revised the manuscript to adopt a more careful interpretation of the findings and to emphasize that the molecular and serological results provide evidence consistent with recent or ongoing arbovirus circulation, rather than definitive proof of active local transmission (L340-358).

**Reviewer’s comment**
L336: USUV RNA detected, by rt-PCR only, no sequencing or virus isolation to corroborate. Please ensure the result is accurately reported

**Response**

We thank the reviewer for this valuable suggestion. We agree that the findings should be reported clearly given the absence of sequence confirmation and virus isolation. We have therefore revised the manuscript to state that USUV RNA was detected by RT-PCR in feather samples, rather than referring to detection of the virus itself. We have also highlighted the lack of sequence confirmation and virus isolation as limitations of the study (L354-359).

**Reviewer’s comment**
L353:optimal cold storage. But could also be stage of infection? i.e. the bird may have been infected some time ago and almost cleared the infection?

**Response**

We thank the reviewer for this comment. We agree that differences in viral detection between feathers and swabs may not fully be explained by storage conditions. The stage of infection at the time of sampling could also have contributed to the observed pattern, as viral RNA may persist longer in feathers after it has become undetectable in swabs. We have revised the Discussion to acknowledge these alternative explanations (L369-383).

**Reviewer’s comment**
L404: Here and more generally, you dont mention the utility of mosquito surveillance to help corroborate your results, obviously you didnt do it here, but it would be useful for future work?

**Response**

We thank the reviewer for this suggestion. We agree that integrating mosquito surveillance with avian surveillance would strengthen evidence for local transmission and improve understanding of arbovirus ecology in the region. We have added his point into the Discussion section to highlight the value of future entomological surveillance to identify circulating viruses, vector species involved, and transmission dynamics at the study locations (L412-416).

**Additional Editor Comments:**

The manuscript is well written, clear, and comprehensive. The study addresses an important topic, and the dataset collected represents a valuable contribution to the understanding of arbovirus circulation in wild bird populations in Africa.

I have only a minor comments related to the interpretation of some findings. In particular, I would suggest slightly tempering the interpretation of the detection of USUV RNA in feathers. While the Discussion is generally careful, some statements (including in the Abstract) may somewhat overextend the implications of RNA detection alone. Given that no infectious virus was isolated, it would be helpful to more clearly distinguish between evidence of viral RNA presence and evidence of active infection or transmission potential.

We thank the Editor for this critical observation. We agree that the detection of viral RNA alone without viral isolation does not constitute definitive evidence of active infection or circulation. Therefore, we have revised the Abstract, Discussion and Conclusion sections to clearly distinguish between USUV RNA and evidence of active infection.

Similarly, the suggestion that migratory birds may contribute to long-distance dispersal of the virus, although plausible and supported by previous literature, could be framed more cautiously in light of the present data.

We thank the Editor for this valuable suggestion. We agree that the present study does not directly demonstrate long-distance dispersal of arboviruses by migratory birds. We have therefore revised the relevant sections of the Discussion to clearly indicate that such dispersal remains a hypothesis supported by previous studies and by the ecology of the sampled species, rather than a direct finding from our work (L351 – 353).

Regarding the use of feathers as a sampling matrix, this is an interesting and potentially important observation. However, the conclusion that feathers may represent a suitable alternative to swabs appears to rely on assumptions about sample storage conditions that are not directly demonstrated. Addressing these points would further strengthen the manuscript and ensure that the conclusions remain fully supported by the data.

We thank the Editor for this important observation. We agree that our study was not designed to directly compare the diagnostic performance of feathers and swabs under different storage conditions. We have therefore revised the Discussion to show that and present feathers as a potentially useful complementary sampling option. Additional text and a reference have been added to acknowledge other studies that have been conducted to demonstrated that for USUV in birds (L375 – 383).
