# Supplementary material for: Shared viral burdens: Molecular evidence of Usutu virus infection and multi-arbovirus exposure in migrant and resident birds at wintering locations in Nigeria
Source: PLoS Negl Trop Dis. 2026 Aug 19;20(8):e0013538. doi: 10.1371/journal.pntd.0013538 (PMC13502912; doi:10.1371/journal.pntd.0013538)
Supplement: Peer review history

# Peer review: Decision Letter 0

**DOI:** 10.1371/journal.pntd.0013538.r001

**Authors:** Camille Lebarbenchon, Abdallah M. Samy

12 Apr 2026

PNTD-D-25-01602

Shared viral burdens: Evidence of active Usutu virus circulation and multi-arbovirus exposure in migrant and resident birds at wintering locations in Nigeria

PLOS Neglected Tropical Diseases

Dear Dr. Atama,

Thank you for submitting your manuscript to PLOS Neglected Tropical Diseases. We sincerely apologize for the delay in the review process. We experienced difficulties in securing reviewers for your manuscript. We have now received one external review, and I have also carefully evaluated the manuscript.

After careful consideration, we feel that it has merit but does not fully meet PLOS Neglected Tropical Diseases's publication criteria as it currently stands. Therefore, we invite you to submit a revised version of the manuscript that addresses the points raised during the review process.

Please submit your revised manuscript by Jun 11 2026 11:59PM. If you will need more time than this to complete your revisions, please reply to this message or contact the journal office at plosntds@plos.org. When you're ready to submit your revision, log on to https://www.editorialmanager.com/pntd/ and select the 'Submissions Needing Revision' folder to locate your manuscript file.

Please include the following items when submitting your revised manuscript:

* A letter that responds to each point raised by the editor and reviewer(s). You should upload this letter as a separate file labeled 'Response to Reviewers'. This file does not need to include responses to any formatting updates and technical items listed in the 'Journal Requirements' section below.

* A marked-up copy of your manuscript that highlights changes made to the original version. You should upload this as a separate file labeled 'Revised Manuscript with Track Changes'.

* An unmarked version of your revised paper without tracked changes. You should upload this as a separate file labeled 'Manuscript'.

If you would like to make changes to your financial disclosure, competing interests statement, or data availability statement, please make these updates within the submission form at the time of resubmission. Guidelines for resubmitting your figure files are available below the reviewer comments at the end of this letter.

We look forward to receiving your revised manuscript.

Kind regards,

Camille Lebarbenchon

Academic Editor

PLOS Neglected Tropical Diseases

Abdallah Samy

Section Editor

PLOS Neglected Tropical Diseases

Shaden Kamhawi

co-Editor-in-Chief

PLOS Neglected Tropical Diseases

orcid.org/0000-0003-4304-636XX

Paul Brindley

co-Editor-in-Chief

PLOS Neglected Tropical Diseases

orcid.org/0000-0003-1765-0002

**Additional Editor Comments:**

The manuscript is well written, clear, and comprehensive. The study addresses an important topic, and the dataset collected represents a valuable contribution to the understanding of arbovirus circulation in wild bird populations in Africa.

I have only a minor comments related to the interpretation of some findings. In particular, I would suggest slightly tempering the interpretation of the detection of USUV RNA in feathers. While the Discussion is generally careful, some statements (including in the Abstract) may somewhat overextend the implications of RNA detection alone. Given that no infectious virus was isolated, it would be helpful to more clearly distinguish between evidence of viral RNA presence and evidence of active infection or transmission potential.

Similarly, the suggestion that migratory birds may contribute to long-distance dispersal of the virus, although plausible and supported by previous literature, could be framed more cautiously in light of the present data. Regarding the use of feathers as a sampling matrix, this is an interesting and potentially important observation. However, the conclusion that feathers may represent a suitable alternative to swabs appears to rely on assumptions about sample storage conditions that are not directly demonstrated.

Addressing these points would further strengthen the manuscript and ensure that the conclusions remain fully supported by the data.

Best regards,

Camille Lebarbenchon

**Journal Requirements:**

If the reviewer comments include a recommendation to cite specific previously published works, please review and evaluate these publications to determine whether they are relevant and should be cited. There is no requirement to cite these works unless the editor has indicated otherwise.

1) Please ensure that the CRediT author contributions listed for every co-author are completed accurately and in full.

At this stage, the following Authors/Authors require contributions: Nnomzie Charles Atama, Joy Akpanta Ishong, Cora M Holicki, Yahkat Barshep, Felicity D Chandler, Anne van der Linden, Marion P G Koopmans, and Reina S Sikkema. Please ensure that the full contributions of each author are acknowledged in the "Add/Edit/Remove Authors" section of our submission form.

The list of CRediT author contributions may be found here: https://journals.plos.org/plosntds/s/authorship#loc-author-contributions

2) We ask that a manuscript source file is provided at Revision. Please upload your manuscript file as a .doc, .docx, .rtf or .tex. If you are providing a .tex file, please upload it under the item type u2018LaTeX Source Fileu2019 and leave your .pdf version as the item type u2018Manuscriptu2019.

3) Please provide an Author Summary. This should appear in your manuscript between the Abstract (if applicable) and the Introduction, and should be 150-200 words long. The aim should be to make your findings accessible to a wide audience that includes both scientists and non-scientists. Sample summaries can be found on our website under Submission Guidelines:

https://journals.plos.org/plosntds/s/submission-guidelines#loc-parts-of-a-submission

4) We do not publish any copyright or trademark symbols that usually accompany proprietary names, eg ©,  ®, or TM  (e.g. next to drug or reagent names). Therefore please remove all instances of trademark/copyright symbols throughout the text, including:

- TM on page: 10.

5) Please upload all main figures as separate Figure files in .tif or .eps format. For more information about how to convert and format your figure files please see our guidelines:

https://journals.plos.org/plosntds/s/figures

6) In the online submission form, you indicated that your data will be submitted to a repository upon acceptance. We strongly recommend all authors deposit their data before acceptance, as the process can be lengthy and hold up publication timelines. Please note that, though access restrictions are acceptable now, your entire minimal dataset will need to be made freely accessible if your manuscript is accepted for publication. This policy applies to all data except where public deposition would breach compliance with the protocol approved by your research ethics board. If you are unable to adhere to our open data policy, please kindly revise your statement to explain your reasoning and we will seek the editor's input on an exemption.

7) Please amend your detailed Financial Disclosure statement. This is published with the article. It must therefore be completed in full sentences and contain the exact wording you wish to be published.

1) State what role the funders took in the study. If the funders had no role in your study, please state: "The funders had no role in study design, data collection and analysis, decision to publish, or preparation of the manuscript."

2) If any authors received a salary from any of your funders, please state which authors and which funders..

If you did not receive any funding for this study, please simply state: u201cThe authors received no specific funding for this work.u201d

8) Some material included in your submission may be copyrighted. According to PLOS's copyright policy, authors who use figures or other material (e.g., graphics, clipart, maps) from another author or copyright holder must demonstrate or obtain permission to publish this material under the Creative Commons Attribution 4.0 International (CC BY 4.0) License used by PLOS journals. Please closely review the details of PLOS's copyright requirements here: PLOS Licenses and Copyright. If you need to request permissions from a copyright holder, you may use the PLOS Content Copyright Permission Form Please respond directly to this email and provide any known details concerning your material's license terms and permissions required for reuse, even if you have not yet obtained copyright permissions or are unsure of your material's copyright compatibility. Once you have responded and addressed all other outstanding technical requirements, you may resubmit your manuscript within Editorial Manager. Potential Copyright Issues:

i) Please confirm (a) that you are the photographer of 1B, or (b) provide written permission from the photographer to publish the photo(s) under our CC BY 4.0 license.

ii) Figures 1A, 1B, and 1D. Please (a) provide a direct link to the base layer of the map (i.e., the country or region border shape) and ensure this is also included in the figure legend; and (b) provide a link to the terms of use / license information for the base layer image or shapefile. We cannot publish proprietary or copyrighted maps (e.g. Google Maps, Mapquest) and the terms of use for your map base layer must be compatible with our CC BY 4.0 license. Note: if you created the map in a software program like R or ArcGIS, please locate and indicate the source of the basemap shapefile onto which data has been plotted. If your map was obtained from a copyrighted source please amend the figure so that the base map used is from an openly available source. Alternatively, please provide explicit written permission from the copyright holder granting you the right to publish the material under our CC BY 4.0 license. If you are unsure whether you can use a map or not, please do reach out and we will be able to help you. The following websites are good examples of where you can source open access or public domain maps: * U.S. Geological Survey (USGS) - All maps are in the public domain. (http://www.usgs.gov) * PlaniGlobe - All maps are published under a Creative Commons license so please cite u201cPlaniGlobe, http://www.planiglobe.com, CC BY 2.0u201d in the image credit after the caption. (http://www.planiglobe.com/?lang=enl) * Natural Earth - All maps are public domain. (http://www.naturalearthdata.com/about/terms-of-use/).

**Reviewers' comments:**

Reviewer's Responses to Questions

**Key Review Criteria Required for Acceptance?**

As you describe the new analyses required for acceptance, please consider the following:

**Methods**

-Are the objectives of the study clearly articulated with a clear testable hypothesis stated?

-Is the study design appropriate to address the stated objectives?

-Is the population clearly described and appropriate for the hypothesis being tested?

-Is the sample size sufficient to ensure adequate power to address the hypothesis being tested?

-Were correct statistical analysis used to support conclusions?

-Are there concerns about ethical or regulatory requirements being met?

Reviewer #1: No issues to report with the methodology

**********

**Results**

-Does the analysis presented match the analysis plan?

-Are the results clearly and completely presented?

-Are the figures (Tables, Images) of sufficient quality for clarity?

Reviewer #1: Results are clearly described and appropriately analysed.

**********

**Conclusions**

-Are the conclusions supported by the data presented?

-Are the limitations of analysis clearly described?

-Do the authors discuss how these data can be helpful to advance our understanding of the topic under study?

-Is public health relevance addressed?

Reviewer #1: No concerns with the conclusions

**********

**Editorial and Data Presentation Modifications?**

Use this section for editorial suggestions as well as relatively minor modifications of existing data that would enhance clarity. If the only modifications needed are minor and/or editorial, you may wish to recommend “Minor Revision” or “Accept”.

Reviewer #1: Minor revision

**********

**Summary and General Comments**

Use this section to provide overall comments, discuss strengths/weaknesses of the study, novelty, significance, general execution and scholarship. You may also include additional comments for the author, including concerns about dual publication, research ethics, or publication ethics. If requesting major revision, please articulate the new experiments that are needed.

Reviewer #1: Thank you for inviting me to review 'Shared viral burdens: Evidence of active Usutu virus circulation and multi-arbovirus exposure in migrant and resident birds at wintering locations in Nigeria' by Atama et al. The manuscript was very interesting, study well designed and very well written. Overall the manuscript warrants publication and used appropriate statistical analyses throughout.

I really only have a couple of very minor edits to suggest:

L41: Should 'was' be 'were'

L105-107: you dont specify that these were sampled in Nigeria, its implied, but might not be obvious

L108: When was SINV detected, if someone reads this paper in 20 years the detection wouldn't be recent, so best to be specific

L112: 253 migratory bird 'species'. Just to clarify that it isn't just a home for 253 birds

L175: You say swabs were shortly stored under 4oC. Can you be more specific, what was the window where they were out of cold storage, how many hours?

L193: We typically use ct 35 as a cut off, due to inability to sequence and corroborate the PCR. Can the authors confirm that the 6 positive USUV feathers were positive on both the screening and confirmatory PCR assays, and can they provide cts for both assays in the results section. This will provide important evidence for one of their main findings. Moreover, if the authors could provide the number of bp each assay should recover that would be beneficial.

L334: The low number of positives and very late cts dont immediately suggest active circulation. These could have been historic infections picked up from other localities prior to sampling in Nigeria. Moreover, given the broad geographic range of these arboviruses, the serological results could also be due to historic exposure in different areas. I agree transmission is likely to occur in Nigeria, but could the authors contextualise these findings appropriately throughout the ms, with some caveats.

L336: USUV RNA detected, by rt-PCR only, no sequencing or virus isolation to corroborate. Please ensure the result is accurately reported

L353:optimal cold storage. But could also be stage of infection? i.e. the bird may have been infected some time ago and almost cleared the infection?

L404: Here and more generally, you dont mention the utility of mosquito surveillance to help corroborate your results, obviously you didnt do it here, but it would be useful for future work?

**********

PLOS authors have the option to publish the peer review history of their article (what does this mean?). If published, this will include your full peer review and any attached files.

If you choose “no”, your identity will remain anonymous but your review may still be made public.

**Do you want your identity to be public for this peer review?**  For information about this choice, including consent withdrawal, please see our Privacy Policy.

Reviewer #1: No

**Figure resubmission:**

While revising your submission, we strongly recommend that you use PLOS’s NAAS tool (https://ngplosjournals.pagemajik.ai/artanalysis) to test your figure files. NAAS can convert your figure files to the TIFF file type and meet basic requirements (such as print size, resolution), or provide you with a report on issues that do not meet our requirements and that NAAS cannot fix.

After uploading your figures to PLOS’s NAAS tool - https://ngplosjournals.pagemajik.ai/artanalysis, NAAS will process the files provided and display the results in the "Uploaded Files" section of the page as the processing is complete. If the uploaded figures meet our requirements (or NAAS is able to fix the files to meet our requirements), the figure will be marked as "fixed" above. If NAAS is unable to fix the files, a red "failed" label will appear above. When NAAS has confirmed that the figure files meet our requirements, please download the file via the download option, and include these NAAS processed figure files when submitting your revised manuscript.

**Reproducibility:**

To enhance the reproducibility of your results, we recommend that authors of applicable studies deposit laboratory protocols in protocols.io, where a protocol can be assigned its own identifier (DOI) such that it can be cited independently in the future. Additionally, PLOS ONE offers an option to publish peer-reviewed clinical study protocols. Read more information on sharing protocols at https://plos.org/protocols?utm_medium=editorial-email&utm_source=authorletters&utm_campaign=protocols

# Author comment: Author response to Decision Letter 1

**DOI:** 10.1371/journal.pntd.0013538.r002

26 Jun 2026

Attachment Submitted filename: Response to Reviewers.docx

# Editor report: Decision Letter 1

**DOI:** 10.1371/journal.pntd.0013538.r003

**Authors:** Camille Lebarbenchon, Abdallah M. Samy

29 Jul 2026

Dear Atama,

We are pleased to inform you that your manuscript 'Shared viral burdens: Molecular evidence of Usutu virus infection and multi-arbovirus exposure in migrant and resident birds at wintering locations in Nigeria' has been provisionally accepted for publication in PLOS Neglected Tropical Diseases.

Before your manuscript can be formally accepted you will need to complete some formatting changes, which you will receive in a follow up email. A member of our team will be in touch with a set of requests.

Please note that your manuscript will not be scheduled for publication until you have made the required changes, so a swift response is appreciated.

IMPORTANT: The editorial review process is now complete. PLOS will only permit corrections to spelling, formatting or significant scientific errors from this point onwards. Requests for major changes, or any which affect the scientific understanding of your work, will cause delays to the publication date of your manuscript.

Should you, your institution's press office or the journal office choose to press release your paper, you will automatically be opted out of early publication. We ask that you notify us now if you or your institution is planning to press release the article. All press must be co-ordinated with PLOS.

Thank you again for supporting Open Access publishing; we are looking forward to publishing your work in PLOS Neglected Tropical Diseases.

Best regards,

Camille Lebarbenchon

Academic Editor

PLOS Neglected Tropical Diseases

Abdallah Samy

Section Editor

PLOS Neglected Tropical Diseases

Shaden Kamhawi

co-Editor-in-Chief

PLOS Neglected Tropical Diseases

orcid.org/0000-0003-4304-636XX

Paul Brindley

co-Editor-in-Chief

PLOS Neglected Tropical Diseases

orcid.org/0000-0003-1765-0002

***********************************************************

# Editor report: Acceptance letter

**DOI:** 10.1371/journal.pntd.0013538.r004

**Authors:** Camille Lebarbenchon, Abdallah M. Samy

Dear Atama,

We are delighted to inform you that your manuscript, "Shared viral burdens: Molecular evidence of Usutu virus infection and multi-arbovirus exposure in migrant and resident birds at wintering locations in Nigeria," has been formally accepted for publication in PLOS Neglected Tropical Diseases.

We have now passed your article onto the PLOS Production Department who will complete the rest of the publication process. All authors will receive a confirmation email upon publication.

The corresponding author will soon be receiving a typeset proof for review, to ensure errors have not been introduced during production. Please review the PDF proof of your manuscript carefully, as this is the last chance to correct any scientific or type-setting errors. Please note that major changes, or those which affect the scientific understanding of the work, will likely cause delays to the publication date of your manuscript. Note: Proofs for Front Matter articles (Editorial, Viewpoint, Symposium, Review, etc...) are generated on a different schedule and may not be made available as quickly.

Soon after your final files are uploaded, the early version of your manuscript will be published online unless you opted out of this process. The date of the early version will be your article's publication date. The final article will be published to the same URL, and all versions of the paper will be accessible to readers.

For Research Articles, you will receive an invoice from PLOS for your publication fee after your manuscript has reached the completed accept phase. If you receive an email requesting payment before acceptance or for any other service, this may be a phishing scheme. Learn how to identify phishing emails and protect your accounts at https://explore.plos.org/phishing.

Thank you again for supporting open-access publishing; we are looking forward to publishing your work in PLOS Neglected Tropical Diseases.

Best regards,

Shaden Kamhawi

co-Editor-in-Chief

PLOS Neglected Tropical Diseases

Paul Brindley

co-Editor-in-Chief

PLOS Neglected Tropical Diseases
